# Lactoferrin and Osteopontin Cooperatively Promote Intestinal Epithelial Maturation in Neonatal Mice by Activating the Brg1/Notch1/Hes1 Pathway

**DOI:** 10.3390/nu17193176

**Published:** 2025-10-08

**Authors:** Wen Zhang, Chuangang Li, Ran Bi, Yao Lu, Yiran Zhang, Chenhong Shi, Ziyu Qiao, Yanan Sun, Juan Chen, Pengjie Wang, Ran Wang, Fazheng Ren, Yixuan Li

**Affiliations:** 1 KKey Laboratory of Functional Dairy, Key Laboratory of Precision Nutrition and Food Quality, Department of Nutrition and Health, China Agricultural University, Beijing 100193, China; zhangwen02919@126.com (W.Z.); biran0429@163.com (R.B.); yiran1119@cau.edu.cn (Y.Z.); shichenhong@cau.edu.cn (C.S.); ziyyqiao@163.com (Z.Q.); 15153515695@163.com (Y.S.); chenjuan@cau.edu.cn (J.C.); wpj1019@cau.edu.cn (P.W.); wangran@cau.edu.cn (R.W.); renfazheng@263.net (F.R.); 2 College of Food Science & Nutritional Engineering, China Agricultural University, Beijing 100083, China; lichuangang1231@126.com (C.L.); lylywhm@126.com (Y.L.)

**Keywords:** intestinal epithelium, lactoferrin, neonatal mice, Notch pathway, osteopontin

## Abstract

**Background/Objectives:** Early life is crucial for infant gut development and intestinal homeostasis. Lactoferrin (LF) and osteopontin (OPN) are bioactive breast milk proteins that are supplemented into infant formula to promote gut development. However, the combined effect of LF and OPN (LOP) on in vivo gut maturation has not been fully elucidated. This study investigated the effects of LF, OPN, and LOP on intestinal epithelium maturation in C57BL/6N mice from postnatal days 7 to 21. **Methods:** 3-day-old pups were assigned to four groups: Control group, LF group: 300 mg/kg LF; OPN group: 300 mg/kg OPN, LOP group: 300 mg/kg of a 1:5 (w/w) mixture of LF and OPN. **Results:** Compared to controls, LOP reduced plasma Diamine Oxidase (DAO) activity by 1.54-fold and D-lactate levels by 1.41-fold, demonstrating greater efficacy than LF or OPN alone in reducing intestinal permeability. LOP also significantly increased intestinal absorptive cells versus controls or single proteins. Mechanistically, LOP promoted directional intestinal stem cell differentiation, increasing jejunal transit-amplifying cells by 1.40-fold in 21-day-old mice. LOP upregulated expression of the Notch pathway target Hes1 by 1.70-fold. Further investigations revealed LOP activated Notch signaling via the transcription factor Brg1. Validation using intestinal organoids and IEC-6 cells confirmed intact OPN within LOP mediates increased Brg1 expression, activating the Notch pathway to direct intestinal stem cell differentiation into absorptive cells. **Conclusions:** Collectively, these findings in neonatal mice suggest that LOP cooperatively enhances intestinal barrier maturation and directs stem cell differentiation via Brg1-Notch signaling, offering potential insights for future research on bioactive protein supplementation in infant nutrition.

## 1. Introduction

The intestinal epithelium fulfills several crucial roles, including nutrient digestion and absorption as well as forming a barrier against intraluminal pathogens, functions that require complete development and maintenance in early life [1], especially during lactation, which is a critical period for the establishment of mature intestinal homeostasis. However, during this period, infant intestinal development is susceptible to external factors that can impair intestinal function, which can result in a predisposition to severe pathologies such as necrotizing enterocolitis (NEC), sepsis, and infectious diarrhea [2]. The diverse functionality of the intestinal epithelium, encompassing absorptive, goblet, Paneth, and enteroendocrine cells, is derived from the proliferation and differentiation of intestinal stem cells (ISCs) in the crypt base. This process, in turn, is orchestrated by key signaling pathways such as Wnt, BMP, and Notch [3]. The Notch signaling pathway is pivotal in directing the cell fate decision of ISCs toward either an absorptive or secretory lineage [4]. The downstream transcription factors of the Notch pathway, particularly the Hes family genes, determine the ultimate fate of stem cells. The cell fate decision between absorptive and secretory lineages is governed by Notch signaling: active Hes1 suppresses Atoh1 to specify absorptive cells, while pathway inhibition shifts the balance toward secretory differentiation. During development, the intestine gradually matures, with early structural development reflecting a balance between cell proliferation and differentiation. Thus, establishing intestinal epithelial homeostasis in early life is essential for subsequent growth and development.

Providing adequate nutritional support to infants after birth is crucial for their intestinal development. Consequently, breast milk is recognized as the superior nutritional source, forming the basis for the global recommendation of exclusive breastfeeding for the initial 6 months. When breastfeeding is suboptimal or infeasible, infant formula serves as the primary alternative nutrition source. Numerous studies have reported that various active components in breast milk are essential for promoting intestinal health. Lactoferrin (LF) and osteopontin (OPN) are among the most important active components in breast milk, and they are permitted to be added to infant formula to support healthy intestinal development. LF, an 80 kDa iron-binding glycoprotein, is abundant in human colostrum and found at moderate concentrations in both human and bovine milk [5]. Early-life LF supplementation is believed to support small intestinal functional development. Bovine lactoferrin (bLF) supplementation regulates intestinal epithelial cell turnover; in vivo, it enhances jejunal villus height and brush border enzyme activity in mice, while in vitro it stimulates the proliferation of Caco-2 and IEC-18 cell lines [6,7]. The study by Buccigrossi V et al. found that human lactoferrin (N-LF) stimulated the growth and proliferation of immature Caco-2 cells at high concentrations and promoted their differentiation at low concentrations [8]. Furthermore, clinical evidence also indicates that LF can enhance intestinal function in infancy and early childhood [9]. OPN is an acidic phosphorylated whey glycoprotein, present in high concentrations in human milk, whereas bovine milk contains comparatively low levels [10]. OPN has been found to enhance the differentiation capacity of Caco-2 cell. OPN contains integrin and CD44 binding sites, and early-life oral intake of bovine OPN has been shown to promote intestinal proliferation and differentiation by upregulating the expression of integrin αvβ3 and CD44 [11].

In recent years, the interactions among various milk-derived proteins have received increasing attention. How LF and OPN collectively stimulate the proliferation and differentiation of intestinal epithelial cells is yet to be determined. Therefore, this study sought to examine whether LF and OPN exerted a synergistic enhancement effect regarding neonatal mouse intestinal epithelium development. The findings will provide a foundation for developing materials that target and regulate early-life intestinal health through LF and OPN.

## 2. Materials and Methods

### 2.1. Materials

Bovine LF: Purity > 95% and 10.5% Fe saturation, purchased from HILMAR INGREDIENTS (Hilmar, CA, USA). Bovine OPN: Purity > 95%, purchased from Arla Foods Ingredients (Videbæk, Denmark). LOP: A combination of LF and OPN at a 1:5 ratio as used in the study.

### 2.2. Animals and Cells

A total of ninety-six 3-day-old male C57BL/6N mouse pups, derived from 64 female and 48 male breeders purchased from Beijing SiPeiFu (Beijing, China) (The sample size was determined based on effect sizes from pilot experiments and previously reported studies [5], with six animals included per group for final analysis), were randomly assigned to four experimental groups. The groups received daily oral gavage for two weeks as follows: Control group: 0.85% NaCl; LF group: 300 mg/kg LF; OPN group: 300 mg/kg OPN, LOP group: 300 mg/kg of a 1:5 (*w*/*w*) mixture of LF and OPN (the ratio was determined based on our prior research [12]). All pups had ad libitum access to breast milk and were housed in a specific pathogen-free (SPF) facility at the Experimental Animal Platform of China Agricultural University. Environmental conditions were maintained at 20–22 °C, 50–60% humidity, and a 12 h light/dark cycle. The animal study protocol was approved by the Ethics Committee for Animal Care of China Agricultural University (Approval No. Aw71503202-5-1, Date: 17 May 2023).

IEC-6 cells (Rat Small Intestine Crypt Epithelial Cells, CRL-1592) were purchased from ATCC (Manassas, VA, USA) and cultured in DMEM supplemented with 10% fetal bovine serum and 1% penicillin-streptomycin at 37 °C in a 5% CO_2_ atmosphere. Cells were passaged every three days and used for subsequent experiments after 3 to 5 passages.

### 2.3. Sample Collection

Pups first received an intraperitoneal injection of sodium pentobarbital (50 mg/kg BW, 1%) for anesthesia. Subsequently, blood was collected via orbital puncture. Serum, obtained by centrifugation (3000× *g*, 15 min, 4 °C), was stored at −80 °C until assayed for DAO and D-lactate. Jejunal tissues were either fixed in 4% paraformaldehyde and embedded in paraffin or flash-frozen at −80 °C for further studies.

### 2.4. H&E and PAS Staining

The fixed jejunum samples were dehydrated, embedded, and sectioned, followed by H&E staining and PAS staining. For H&E staining, slices were deparaffinized by immersing them in xylene and a graded ethanol series. Tissue sections were stained with hematoxylin (Leagene, Beijing, China) for 30 s and subsequently counterstained with eosin (Solarbio, Beijing, China) for 1 min. Goblet cells were specifically identified using PAS staining. Using a glycogen staining kit (Leagene, Beijing, China), the tissue was incubated with periodic acid for 5 min and then stained with Schiff reagent for 10 min. Hematoxylin was then applied to stain the cell nuclei. All stained slices were then dehydrated and mounted with neutral balsam, and finally were observed.

### 2.5. Immunofluorescence (IF) and Immunohistochemistry (IHC)

Following deparaffinization, tissue slices underwent antigen retrieval by microwave heating for 25 min in sodium citrate buffer (pH 6.0). After cooling to room temperature, endogenous peroxidase activity was quenched with 3% H_2_O_2_. The slices were then permeabilized with 1% Triton-X 100 and blocked with 10% normal goat serum prior to an overnight incubation with the primary antibody at 4 °C. slices were incubated with a fluorophore-conjugated secondary antibody and subsequently counterstained with DAPI (Beyotime, Shanghai, China) to visualize nuclei. For immunohistochemistry (IHC), following primary antibody incubation, a horseradish peroxidase (HRP)-labeled goat anti-rabbit IgG polymer (ZSGB-BIO, Beijing, China) was applied for 20 min at room temperature. The DAB (ZSGB-BIO, China) chromogenic solution, diluted at 1:50, was added until brown positive cells were observed under a microscope. The reaction was terminated by transfer of the slices to distilled water, followed by nuclear staining with hematoxylin. Finally, the slices were mounted for observation under a microscope (Leica, Wetzlar, Germany). Following incubation with the primary antibodies listed below: anti-**Alpi** (Invitrogen, Carlsbad, CA, USA), anti-**Olfm4** (Cell Signaling Technology, Danvers, MA, USA), anti-**Pcna** (Santa Cruz, CA, USA), anti-**Hes1** (Santa Cruz, CA, USA), and anti-**Notch1** (Santa Cruz, CA, USA). Alexa Fluor 488/594-conjugated anti-rabbit/rat IgG (H + L) secondary antibodies (Abcam, Cambridge, UK) were employed. All images were visualized and acquired using laser scanning confocal microscopy (ZEISS, Oberkochen, Germany).

### 2.6. Western Blotting

The jejunum tissues were washed once in phosphate-buffered saline (PBS) and dissolved with RIPA lysis buffer containing penylmethylsulfonyl fluoride (PMSF). The supernatants were harvested after the process of fragmentation and centrifugation. The protein concentration was quantified using a BCA protein assay kit (Beyotime, Shanghai, China). Protein samples mixed with loading buffer were separated on SDS-PAGE gels and then electroblotted onto polyvinylidene fluoride (PVDF) membranes followed by blocking with 5% skim milk for 2 h. Then, the membranes were incubated with anti-**Brg1** (Santa Cruz, CA, USA), **Notch1** (Santa Cruz, CA, USA), **Dll4** (Proteintech, Wuhan, China), **Nrf2** (Proteintech, Wuhan, China), **Hes1** (Santa Cruz, CA, USA) and **β-actin** (Cell Signaling Technology, **MA**, USA) overnight at 4 °C. After washing with PBST, the membranes were incubated at room temperature for 2 h with the anti-rabbit IgG or anti-mouse IgG horseradish peroxidase secondary antibodies. Finally, the blot signals were detected using X-ray (Amersham Imager 600, General Electric Company, Boston, MA, USA). Band intensities were analyzed semi-quantitatively using densitometry with ImageJ software (Fiji version).

### 2.7. In Vitro Simulated Protein Digestion

Based on the methodology described by Moheb Elwakiel et al. [13]. and our previous work [14], in vitro simulated gastrointestinal digestion of LF and OPN was performed. Briefly, 200 mg of LF, OPN, or LOP was dissolved separately in 20 mL of PBS, with the pH pre-adjusted to 4.0, and stirred until complete dissolution was achieved. The pH was further verified and adjusted to 4.0 using NaOH or HCl as necessary. Then, 200 U/mL of pepsin was added, and the mixture was incubated in darkness at 37 °C in a shaking incubator at 200 rpm for 1 h. After simulated gastric digestion, the pH was raised to 7.0 using 1 M NaOH to obtain simulated gastric chyme. A 10 mL aliquot of the simulated gastric chyme was subsequently subjected to simulated intestinal digestion by adding 8.33 U/mL of porcine trypsin. The mixture was incubated again under the same conditions (37 °C, 200 rpm, dark) for 1 h. Finally, to terminate enzymatic activity, the sample was heated in a 95 °C water bath for 10 min and stored at −20 °C for further analysis.

### 2.8. Crypt Isolation and Organoid Culture

The jejunal segment was rinsed with pre-cooled PBS, then longitudinally cut open, and the intestinal villi were scraped off using a coverslip. The remaining tissue was repeatedly washed with pre-cooled PBS until no visible precipitate remained. The intestinal tissue was then cut into 0.5 cm fragments and digested with 5 mM EDTA on a shaker at 4 °C and 100 rpm for 15 min. After removing the supernatant, the intestinal tissue was vigorously shaken in PBS and passed through a 70 μm cell strainer to collect crypts. This step was repeated twice. The collected filtrate was centrifuged at 200 rpm for 5 min, the supernatant was discarded, and the crypts were resuspended in 10 mL of DMEM/F12. A 10 μL sample of the suspension was observed under a microscope to check the extraction of crypts. The remaining suspension was centrifuged, and the crypts were resuspended in a mixed matrixgel medium (GM: Matrigel = 3:7). The culture was maintained at 37 °C with 5% CO_2_, and passaged every 3 days.

### 2.9. Cell Viability Assay

Cells were scraped from the culture flask and seeded into 96-well plates. Four concentrations of LF, OPN, and LOP were set at 50, 100, 500, and 1000 μg/mL, respectively. Once the cell density in the 96-well plates reached 70%, the medium was replaced with a fresh basal medium, and different concentrations of test proteins (LF/OPN/LOP) were added. After 24 h of treatment, a basal medium containing 10% CCK8 was added. The color of the medium in the 96-well plates was observed every half hour, and the incubation time was determined based on the actual number of cells. A microplate reader was used to measure the absorbance at 450 nm in each well following the incubation period.

### 2.10. EDU Staining

Following seeding on coverslips in 24-well plates, cells were treated for 24 h with LF, OPN, or LOP. A preheated (37 °C) EdU working solution was then added to the wells for a 2 h incubation. Subsequently, the medium was aspirated and cells were fixed with 4% paraformaldehyde. After three washes, permeabilization was performed with 0.5% Triton X-100 for 20 min. Following another wash, a Click reaction solution was applied for 30 min in the dark. Finally, nuclei were counterstained with DAPI and imaged by laser scanning confocal microscopy (ZEISS, Oberkochen, Germany).

### 2.11. Cell Cycle Assay

Cell cycle was analyzed by flow cytometer (Beckman, Brea, CA, USA). Following a wash with pre-cooled PBS and centrifugation, cells were gently resuspended in 70% ethanol and fixed at −20 °C for 2 h. Subsequently, the fixed cells were pelleted by centrifugation (500× *g*, 5 min, 4 °C) and washed once with PBS. In the dark, 0.5 mL of propidium iodide staining solution (prepared with 100 μL staining buffer + 5 μL propidium iodide + 2 μL RNaseA) was added. The cells were incubated in a water bath at 37 °C for 30 min in the dark, and the reaction was then stopped on ice.

### 2.12. Statistical Analysis

All statistical analyses were performed using IBM SPSS Statistics 27. Continuous variables are expressed as mean ± SD. Inter-group comparisons were made by one-way ANOVA with Tukey’s post hoc test, considering a *p*-value < 0.05 as statistically significant. All figures were generated with GraphPad Prism 9.

## 3. Results

### 3.1. LOP Contributes to the Maintenance of Intestinal Epithelial Integrity in Neonatal Mice

**Figure 1:**
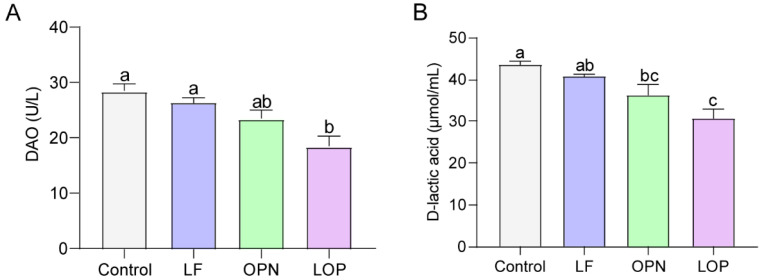
The combined effects of LF and OPN on intestinal permeability in 21-day-old mice. (**A**) Serum DAO activity. (**B**) Serum D-lactate levels. Data are presented as mean ± SD (*n* = 6). Significant differences, determined by one-way ANOVA, are denoted by different superscript letters (a, b, c) (*p* < 0.05).

**Figure 2:**
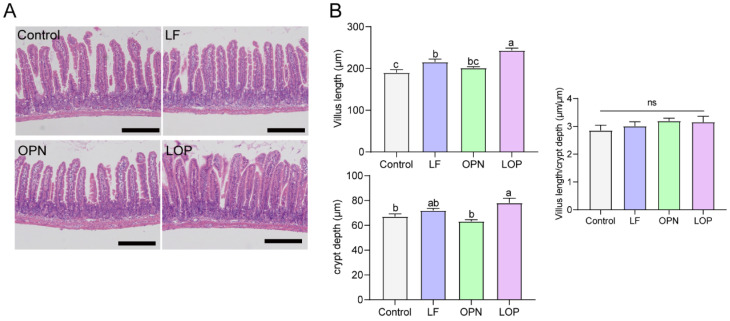
The combined effects of LF and OPN on intestinal structure in 21-day-old mice. (**A**) Representative images of HE-stained jejunal epithelium in mice (10×, scale bar = 200 µm). (**B**) Villus length, crypt depth, and the ratio of villus length to crypt depth. Data are presented as mean ± SD (*n* = 6). Significant differences, determined by one-way ANOVA, are denoted by different superscript letters (a, b, c) (*p* < 0.05); ns: not significant.

Throughout the experimental period, a small number of mice died as a result of intragastric administration procedures. The remaining animals exhibited no abnormal conditions. On postnatal day (PND) 3, neonatal mice were randomly assigned to experimental groups. Oral gavage was initiated on PND 7 and maintained for a duration of two weeks. Blood samples were collected on PND 14 and 21 for the determination of DAO and D-lactate levels to evaluate epithelium integrity. The results, shown in Figure 1A,B, indicated that D-lactate levels and DAO activity decreased in the serum of 21-day-old mice following protein intervention. Compared to the control group, LF had no significant effect, while OPN significantly lowered D-lactate levels in serum. LOP significantly decreased both DAO activity by 1.54-fold (*p* < 0.05) and D-lactate levels by 1.41-fold, demonstrating a superior effect in mitigating intestinal permeability over LF alone. The intestinal epithelium structure is formed by various intestinal epithelial cells. To clarify the effects of LF and OPN on the maturation of intestinal epithelial cells, we conducted statistical analyses of villus height and crypt depth using H&E staining. As shown in Figure 2A,B, the crypt depth in the LOP group was significantly increased by 1.16-fold compared to the control group (*p* < 0.05). Both the LF and LOP treatment groups exhibited significant differences in villus height compared to the control group. Notably, the LOP group showed a significantly higher villus height than the LF group (1.28-fold, *p* < 0.05). The morphometric analysis of intestinal architecture indicated comparable villus height to crypt depth ratios across all experimental groups. Further examination of 14-day-old mice demonstrated increased crypt depth in the LF, OPN, and LOP treatment groups relative to controls, with the most pronounced enhancement observed in the LOP group (Figure S1A,B).

### 3.2. LOP Increases the Population of Intestinal Absorptive Cells in Neonatal Mice

**Figure 3:**
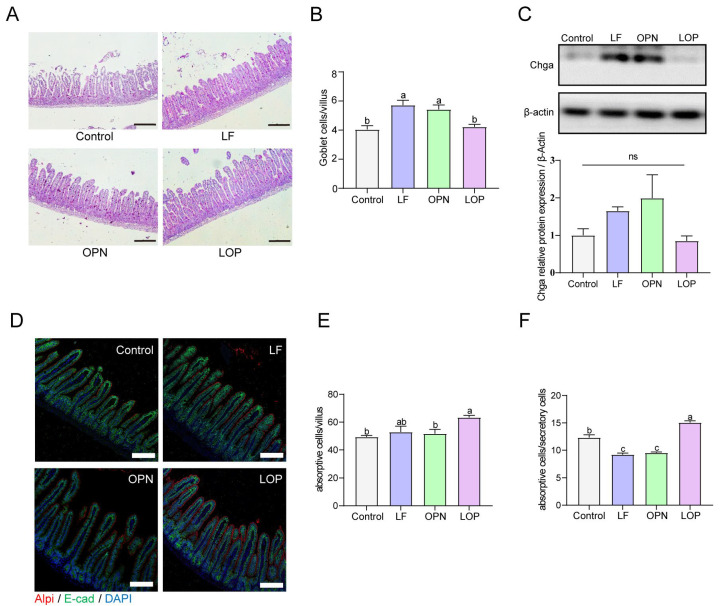
Effect of LF in combination with OPN on intestinal epithelial cells composition in 21-day-old mice. (**A**,**B**) Representative bright-field images and quantification of PAS staining used to indicate goblet cells (10×, scale bar = 200 µm). (C) The relative expression level of chga at the protein level is used to indicate the level of intestinal endocrine cells. (**D**–**F**) Representative immunofluorescence images of absorptive cells (Alpi+) and quantification in each villus. (10×, scale bar = 100 µm). (**F**) The ratio of absorptive cells to secretory cells. Data are presented as mean ± SD (*n* = 6). Significant differences, determined by one-way ANOVA, are denoted by different superscript letters (a, b, c) (*p* < 0.05); ns: not significant.

The integrity of the intestinal epithelium is primarily maintained by epithelial cells, which consist of absorptive and secretory cells. Among them, absorptive cells are dominant, accounting for approximately 90% [15]. To quantify secretory cell populations in the small intestine, enteroendocrine cells were evaluated via Western blot analysis, while goblet cells in jejunal villi were enumerated using PAS staining. As demonstrated in Figure 3A–C, LF supplementation resulted in a significant elevation in enteroendocrine and goblet cell counts relative to the control group. A similar increase in goblet cell numbers was also observed following OPN treatment. However, LOP did not affect the increase in the number of secretory cells. Alkaline phosphatase, a brush border enzyme expressed by absorptive enterocytes, serves as a reliable marker for this predominant intestinal epithelial cell type. As shown in Figure 3D–F, Neither LF nor OPN treatment significantly altered the abundance or functional capacity of absorptive cells relative to the control condition, while LOP significantly increased the number of absorptive cells expressing Alpi in the intestines of pups by 1.28-fold.

### 3.3. LOP Promotes the Proliferation of Transit-Amplifying Cells in Crypts

**Figure 4:**
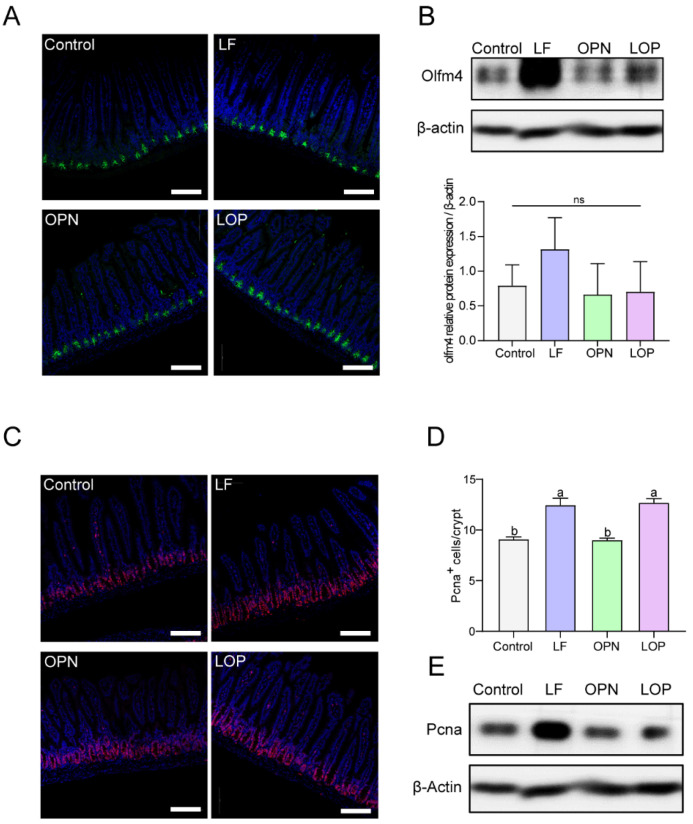
Effect of LF in combination with OPN on intestinal stem cell activation in 21-day-old mice. (**A**) Representative fluorescence images of intestinal stem cells (Olfm4^+^) (10×, scale bar = 100 µm). (**B**) Western blot analysis of **Olfm4** expression in crypts. (**C**,**D**) Representative fluorescence images and quantitative analysis of transit-amplifying cells (**Pcna^+^**) in crypts (10×, scale bar = 100 µm). (**E**) Western blot analysis of **hes1** expression in crypts. Data are shown as mean ± SD, *n* = 3. Data are presented as mean ± SD (*n* = 6). Significant differences, determined by one-way ANOVA, are denoted by different superscript letters (a, b, c). (*p* < 0.05); ns: not significant.

To evaluate the impact of LOP on intestinal stem cells, **Olfm4**—a marker of activated stem cells—was localized within crypt structures via IF staining, while its expression levels were assessed by Western blot. We found that the effects of LF and OPN on the number of activated stem cells in 21-day-old mice were not significant. The expression levels of **Olfm4** in the OPN and LOP groups were similar to those in the control group, while the LF group exhibited higher **Olfm4** expression levels (Figure 4A,B). The same result was also found in 14-day-old mice (Figure S2A,B). We further analyzed the proliferation of stem cells to clarify the effects of LOP on the quantity and function of intestinal Transit-Amplifying (TA) cells, using IF staining to localize the proliferation marker **Pcna**. Both LF and LOP administration significantly elevated the abundance of TA cells in murine intestinal crypts compared to control and OPN groups, showing increases of 1.38-fold (*p* < 0.05) and 1.40-fold (*p* < 0.05), respectively. Moreover, in 14-day-old mice, OPN and LOP also markedly enhanced TA cell numbers (Figure S2C,D).

### 3.4. LOP Promotes the Differentiation of ISCs Toward an Absorptive Lineage Through Activation of the Notch Signaling Pathway

**Figure 5:**
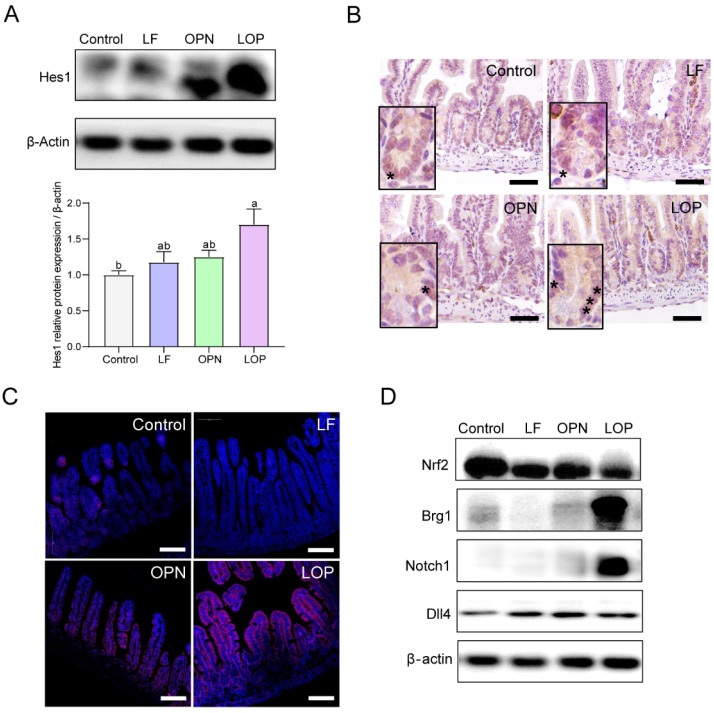
Effect of LF in combination with OPN on Notch signaling pathway in 21-day-old mice. (**A**,**B**) Western blot analysis and representative IHC images of the target protein **Hes1** in the jejunum. The asterisk “*” indicates the cells marked with "hes1" (20×, scale bar = 100 µm). (**C**) Immunostaining images showing the expression of **Notch1** receptor in the jejunum (10×, scale bar = 100 µm). (**D**) Western blot analysis of the transcription factors **Nrf2** and **Brg1**, **Notch1** receptor, and **Dll4** ligand in the Notch pathway. Data are presented as mean ± SD (*n* = 6). Significant differences, determined by one-way ANOVA, are denoted by different superscript letters (a, b, c) (*p* < 0.05).

To investigate the mechanistic basis of LOP-driven directional differentiation of ISCs, we examined the Notch signaling pathway—a key regulator of stem cell fate specification. As shown in Figure 5A,B, the protein expression level of Hes1 in the LOP group was higher than that in the Control, LF, and OPN groups, and the same results were observed in 14-day-old mice (Figure S3A,B). Interestingly, OPN also promoted **Hes1** expression in 14-day-old mice, while no statistically significant differences were observed relative to the control and LF groups.

Next, we examined the receptors and ligands that influence **Hes1** expression and found that LOP increased the expression of the **Notch1** receptor in the Notch signaling pathway, but had no effect on the expression of the **Dll4** ligand. We then analyzed the transcription factors **Brg1** and **Nrf2**, which regulate **Notch1** expression, and found that LOP did not affect **Nrf2** expression but upregulated **Brg1** expression (Figure 5C,D). Therefore, this study suggests that LOP may influence the Notch pathway by activating the transcription factor **Brg1** for **Notch1**.

### 3.5. Evaluation of LOP-Induced Modulation of Notch Signaling Target Proteins in IEC-6 Cells

**Figure 6:**
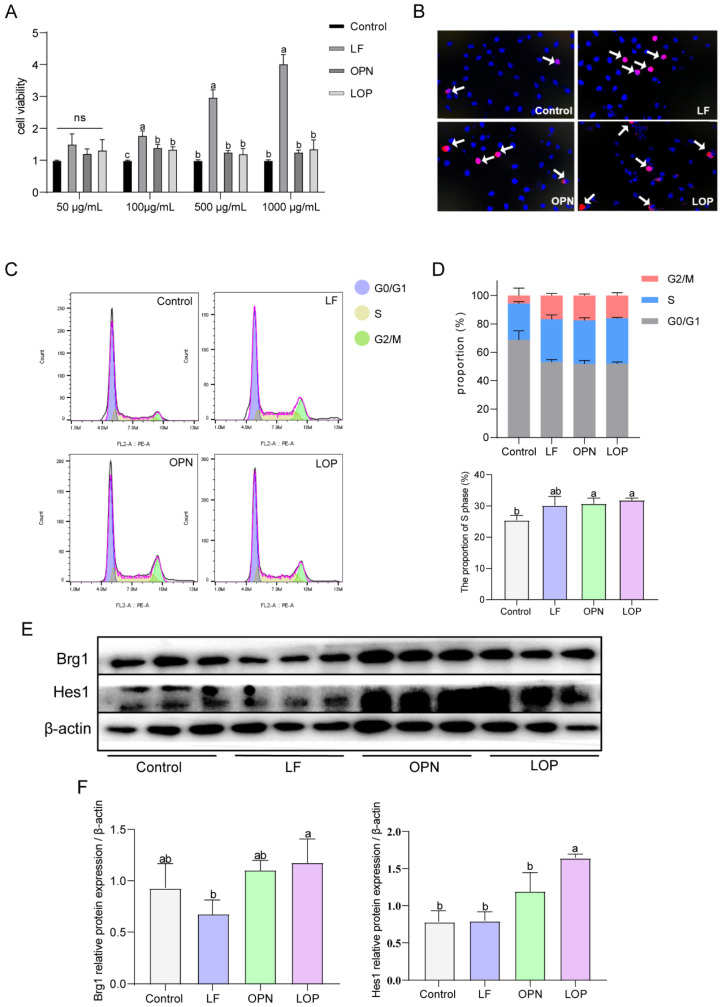
Effect of LF in combination with OPN on the Notch signaling pathway in IEC-6 cells. (**A**) Viability of IEC-6 cells after treatment with different concentrations of LF, OPN, and LOP. (**B**) Proliferation capacity of IEC-6 cells characterized by EDU staining. The arrow indicates the proliferating cells. (**C**) Changes in the cell cycle of IEC-6 cells after treatment with complete LF, OPN, and LOP. (**D**) Proportion of cells in each phase of the cell cycle and the proportion of S-phase cells under different treatments. (**E**,**F**) Western blot analysis of key Notch signaling proteins Hes1 and Brg1 in IEC-6 cells after administration with complete LF, OPN, and LOP. Data are presented as mean ± SD (*n* = 6). Significant differences, determined by one-way ANOVA, are denoted by different superscript letters (a, b, c) (*p* < 0.05); ns: not significant.

Previous studies confirmed that LF largely resists gastric digestion, but no intact form of OPN remains after intestinal digestion. Interestingly, LOP resists gastric digestion and partially resists intestinal digestion [14]. This study employed organoid models to investigate the effects of digested proteins on their morphology in vitro. The results demonstrated that digested LF significantly increased the number of cystic organoids. In contrast, digested OPN did not significantly affect the number of either cystic or budding organoids, and the digested LOP produced effects similar to those of LF. (Figure S4A,D). Based on the previous results of protein digestion, this study suggests that the synergistic effects were likely due to the intact forms of OPN and LF. To validate this hypothesis, we evaluated the cell proliferation and the activation status of the Notch signaling pathway by using the complete protein in the IEC-6 cell model. As shown in (Figure 6A), at a protein concentration of 100 μg/mL, LF significantly increased the viability of IEC-6 cells, and the cell viability in the OPN and LOP groups also showed significant differences compared to the control group. The experimental concentration was set at 100 μg/mL. EDU staining coupled with flow cytometry demonstrated that LF, OPN, and LOP all enhanced S phase entry in IEC-6 cells (Figure 6B–D).

To evaluate the expression of target protein and transcription factors within the Notch signaling cascade, we measured the expression levels of **Hes1** and **Brg1**. As shown in Figure 6E,F, LF alone did not increase the expression of Notch pathway target proteins or the **Notch1** transcription factor **Brg1**. Compared to the control group, OPN alone increased the expression of both **Brg1** and **Hes1**, while LOP significantly increased the expression of **Hes1** along with **Brg1**. Thus, we speculated that the synergistic effect of LF and OPN was fundamentally dependent on the intact form of OPN. The observed effect was driven by Notch pathway activation, which was facilitated by upregulation of the transcription factor **Brg1**.

## 4. Discussion

Existing evidence supports a high-affinity interaction between LF and OPN, which are naturally present in breast milk. This complex exhibits higher bioactivity compared to the individual proteins [16,17,18]. In vitro studies have shown that the LF-OPN mixture promotes the proliferation of intestinal epithelial cells [19]. However, no in vivo studies have yet explored whether this combination can facilitate the maturation of the intestinal epithelial structure and elucidated its underlying mechanisms. Our previous research revealed that, compared to individual proteins, LOP is more capable of attenuating LPS-induced intestinal barrier damage [12]. This study further confirmed that in early life, LOP also displayed a stronger effect in promoting the maturation of the intestinal epithelial structure. Literature indicates that early-life intervention with LF can reduce intestinal permeability and increase the villus height in piglets’ small intestine [20]; OPN has also been shown to promote intestinal barrier integrity, a finding consistent with our study’s results [11]. This study found that both LF and OPN, when used individually, can promote the integrity of the intestinal epithelium, which is in line with the literature. Interestingly, the combined intervention of LOP showed superior effects, being able to reduce intestinal permeability and increase villus height and crypt depth, confirming our hypothesis that the combination promoted the maturation of the intestinal epithelium. Further analysis revealed that LOP significantly increased the population of absorptive cells. This expansion in cell numbers and villus architecture indicates an enhancement of the intestinal absorptive surface area and functional capacity. Compared to adults, infants have a higher metabolic rate and energy requirements [21], indicating that LOP can better meet the nutritional needs of infants and young children, thereby accelerating their intestinal maturation.

The maintenance and turnover of the intestinal epithelium are driven by the activation, expansion, and terminal differentiation of ISCs [22]. Studies have shown that OPN has the function of increasing the abundance of TA cells in the crypts of the jejunum [11]. This study found that OPN could increase TA cells in 14-day-old mice, which is similar to the results in the literature. However, OPN did not significantly alter the abundance of TA cells in 21-day-old mice, which is speculated to be due to the increased digestive capacity of the mice, leading to a gradual weakening of OPN’s impact on the intestines. Additionally, LF enhanced stem cell proliferation and up-regulated **Pcna** expression. This effect was consistently observed in the jejunum of 21-day-old mice, aligning with previously reported findings. LOP had no effect on the number of ISCs but was able to increase the TA cells, indicating that the combined use of LF and OPN could compensate for the weakened effect of OPN due to the increased digestive capacity of the mice.

To investigate the mechanisms by which LOP promotes epithelial cell proliferation and differentiation, we focused on the canonical Notch signaling pathway, which plays a central role in determining ISCs fate. Notch signaling is essential for both maintaining ISC self-renewal and guiding their lineage specification. Activation of Notch signal enhances the proliferation of ISCs, and promotes intestinal progenitor cells differentiation into absorptive lineage cells [4,23,24], which constitute the predominant cell type within the intestinal epithelium [25,26]. This study found that in the intestines of 14-day-old mice, both OPN and LOP could increase the expression of **Hes1**, which corresponded to the crypt depth statistics in this study, suggesting that the Notch pathway at this time was functioning to increase the reserve of the stem cell pool. In the intestines of 21-day-old mice, only LOP had a significant impact on the expression of **Hes1**, which corresponded with the previous results of this study: only LOP could increase the number of absorptive cells per villus and promote the directed differentiation of stem cells. Furthermore, this study found that LOP promotes the increased expression of the **Notch1** receptor and its transcription factor **Brg1**. In addition, the expression of **Dll4**, a classical ligand of **Notch1** [23], was detected, but no significant differences were seen among the treatments. However, the expression of the target protein **Hes1** was significantly elevated. We speculate that the activation of the Notch signaling pathway by LOP may not depend on the upregulation of **Dll4** expression. Instead, LOP likely stimulate the Notch pathway via **Brg1**, thereby inducing **Hes1** transcription; this drives the commitment of ISCs to an absorptive fate and hastens epithelial maturation. We acknowledge that this study did not examine all Notch ligands (such as **Dll1** and **Jagged1**), and thus we cannot completely rule out the possibility that other ligands may mediate the activation of **Notch1** by LOP. Future studies should comprehensively analyze the impact of LOP on the Notch pathway.

Preliminary findings indicated that in the intestines of 14-day-old mice with poor digestive capacity, OPN could increase the expression of **Hes1**; whereas in the intestines of 21-day-old mice with strong digestive capacity, OPN had no significant effect on the expression of **Hes1**. Thus further in vitro verification is needed to determine whether the digested products of LOP exert their biological functions. Previous research found that LF can resist gastrointestinal digestion; OPN is completely digested after passing through the intestine; LOP can resist gastric digestion and partially resist intestinal digestion, When administered together, LF prolongs the intestinal retention of OPN, thereby facilitating the presence of intact OPN within the small intestine [14]. Furthermore, this study found that digestion of LF, OPN, and LOP all increased the number of vesicle-like organoids, but there were no significant differences, and all three treatment groups had no significant effect on the number of budding organoids; therefore, this study speculated that OPN needs to be in its intact form to exert its function. Under physiological conditions, LF carries a positive charge, facilitating its interaction with negatively charged molecules [27,28]. As OPN is negatively charged at physiological pH, electrostatic attraction to cationic LF is expected. We therefore propose that the observed synergy arises from LF-facilitated transcytosis of OPN across the intestinal epithelium, which elevates its luminal concentration and amplifies downstream signaling. In order to confirm our speculation, we further employed the IEC-6 cell model to verify the mechanism of action. Consistent with the results from animal experiments, intact LF also had no effect on the expression of **Hes1** or the activation of the Notch pathway in cells. In contrast, not only did combined OPN significantly upregulate the expression of **Hes1**, but intact OPN alone also had a significant impact on upregulating **Hes1** expression. Although OPN and LOP had no significant effect on **Brg1** expression, there is still a trend towards upregulation compared to the control group. Therefore, the synergistic action between LF and OPN is contingent upon the presence of intact OPN. This effect is mediated through the upregulation of **Brg1**, leading to the activation of the Notch pathway. It should be noted that the IEC-6 cell line has inherent limitations and cannot fully recapitulate the diverse composition and functional maturity of primary human intestinal epithelial cells in vivo. Therefore, while our findings in IEC-6 cells provide valuable mechanistic insights into the role of OPN in activating Brg1–Notch signaling, these results should be interpreted within the constraints of the model system. Future studies should employ models that more accurately represent human intestinal physiology, such as intestinal organoids or in vivo experiments, to further validate and extend these findings.

Interestingly, while the administration of LF or OPN alone increased the number of goblet cells, their combination (LOP) did not result in a further increase in secretory cell populations. This phenomenon may be explained by the observed activation of the Brg1–Notch signaling pathway induced by the combined treatment. Notch activation is known to suppress secretory lineage differentiation, thereby promoting the commitment of stem cells toward the absorptive cell lineage [29]. These results indicate that the effect of LOP is not simply additive but represents a biologically synergistic interaction between LF and OPN. The underlying mechanism may involve enhanced retention of OPN within the LOP complex due to the presence of LF, allowing OPN to remain intact and persistently activate the Notch pathway in the small intestine. This hypothesis was supported by subsequent in vitro experiments in IEC-6 cells, where OPN treatment alone significantly up-regulated the expression of HES1 and Brg1, mirroring the effects observed with the LOP combination. Moreover, previous studies have also reported the role of OPN in Notch pathway activation [30], which is consistent with and reinforces the findings of this study.

Furthermore, it is important to acknowledge that the intestinal epithelium functions within a complex microenvironment. Factors such as the gut microbiota, their metabolites (e.g., short-chain fatty acids), and mucosal immune cells are all known to interact with and influence epithelial development and function [31]. This study did not assess changes in the gut microbial composition, SCFA levels, or mucosal cytokine profiles following LOP intervention. Therefore, we cannot rule out the possibility that alterations in the gut microenvironment may also contribute to—or be influenced by—the effects of LOP on epithelial maturation. Investigating the impact of LOP on gut microbiota–host interactions represent an important direction for future research, aiming to provide a more comprehensive understanding of the mechanisms through which LOP promotes intestinal epithelial maturation.

## 5. Conclusions

**Figure 7:**
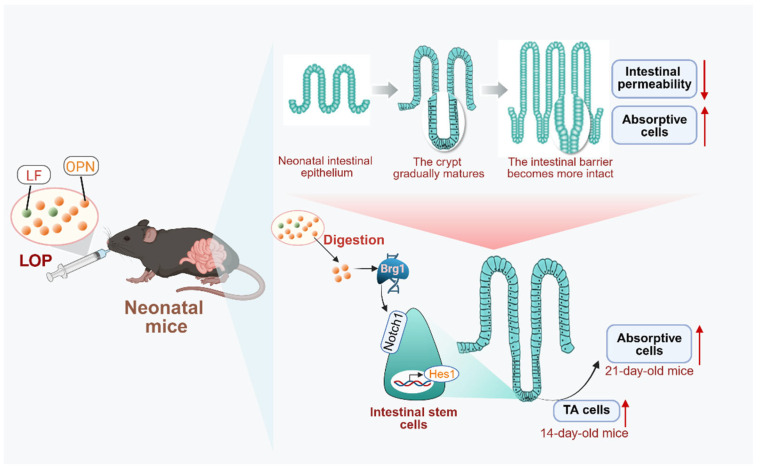
Mechanism by which LF combined with OPN (LOP) promotes intestinal epithelial maturation. LOP upregulates the expression of the transcription factor **Brg1**, which subsequently activates Notch signaling and promotes the transcription of its downstream target gene, **Hes1**. This cascade drives the directional differentiation of intestinal stem cells into absorptive epithelial cells. This regulation exhibits time-dependent characteristics: an increase in TA cells at day 14 provides a cellular reservoir for subsequent differentiation, while a significant increase in mature absorptive cells is observed at day 21. These cellular changes are accompanied by reduced intestinal barrier permeability and improved structural integrity, ultimately leading to the functional maturation of the intestine.

In summary, this study, for the first time, demonstrated that the combined use of LF and OPN, compared to either alone, more effectively promoted the development and maturation of the intestinal epithelial structure, and also revealed that LOP activated the **Brg1/Notch1/Hes1** pathway to promote the directed differentiation of intestinal stem cells into absorptive cells, and intact OPN was the underlying reason (Figure 7). However, how OPN interacts with intestinal stem cells to increase **Brg1** expression and thereby activate the Notch pathway requires further clarification. Collectively, these findings in neonatal mice suggest that LOP cooperatively enhances intestinal barrier maturation and directs stem cell differentiation via Brg1-Notch signaling. These preclinical data highlight the potential of LF and OPN interactions for further investigation, which may inform future studies on bioactive protein supplementation in infant nutrition.

## Data Availability

The original contributions presented in this study are included in the article/Supplementary Materials. Further inquiries can be directed to the corresponding author.

## Supplementary Materials

The following supporting information can be downloaded at https://www.mdpi.com/article/10.3390/nu17193176/s1, Figure S1: The combined effects of LF and OPN on intestinal structure in 14-day-old mice. (A) Representative images of HE-stained jejunal epithelium (10×, scale bar = 200 µm). (B) Villus length, crypt depth, and the ratio of villus length to crypt depth. Data were shown as mean ± SD, *n* = 6. Statistical significance of the differences was determined using one-way ANOVA. Different superscript letters (a, b, c) indicated statistically significant differences (*p* < 0.05). Figure S2: Effects of LF in combination with OPN on intestinal stem cell activation in 14-day-old mice. (A) Representative fluorescence images of intestinal stem cells (Olfm4^+^) (10×, scale bar = 100 µm). (B) Western blot analysis of Olfm4 expression in crypts. (C) Representative fluorescence images and quantitative analysis of transit-amplifying cells (Pcna^+^) in crypts (20×, scale bar = 100 µm). (D) Western blot analysis of Pcna expression in crypts. Data were shown as mean ± SD, *n* = 3. The significance of differences among multiple groups was evaluated using one-way ANOVA. a, b, and c represent significant differences (*p* < 0.05). Figure S3: Effects of LF in combination with OPN on HES1 expression in 14-day-old mice. (A) Western blot analysis of Hes1 protein expression. (B) Representative images of the target protein Hes1 in the jejunum (20×, scale bar = 100 µm). Data were shown as mean ± SD, *n* = 3. The significance of differences among multiple groups was evaluated using one-way ANOVA. a, b, and c represent significant differences (*p* < 0.05). Figure S4: The effects of LF, OPN, and LOP on organoid morphology after in vitro digestion. (A) Representative images of intestinal organoids treated with digested proteins for 3 days. (B) Organoid forming efficiency. (C) Budding rate. (D) spherical rate. Data were shown as mean ± SD, *n* = 3. The significance of differences among multiple groups was evaluated using one-way ANOVA. a, b, and c represent significant differences (*p* < 0.05).

## Abbreviations

LF	Lactoferrin
OPN	Osteopontin
LOP	Combination of Lactoferrin and Osteopontin
DAO	Diamine Oxidase
ISCs	Intestinal stem cells
IEC-6	Rat small intestine crypt epithelial cells
H&E staining	Hematoxylin and eosin staining
NEC	Necrotizing Enterocolitis
PAS staining	Periodic acid-schif staining
IF	Immunofluorescence
IHC	Immunohistochemistry
PBS	Phosphate-buffered saline
PMSF	Penylmethylsulfonyl Fluoride
PVDF	Polyvinylidene Fluoride
TA cells	Transit-Amplifying cells
