# Supplementary material for: Lactoferrin and Osteopontin Cooperatively Promote Intestinal Epithelial Maturation in Neonatal Mice by Activating the Brg1/Notch1/Hes1 Pathway
Source: Nutrients. 2025 Oct 8;17(19):3176. doi: 10.3390/nu17193176 (PMC12525966; doi:10.3390/nu17193176)
Supplement: Supplementary file 1 [file nutrients-17-03176-s001.zip › nutrients-3885446-supplementary.pdf]

Supplementary Figures

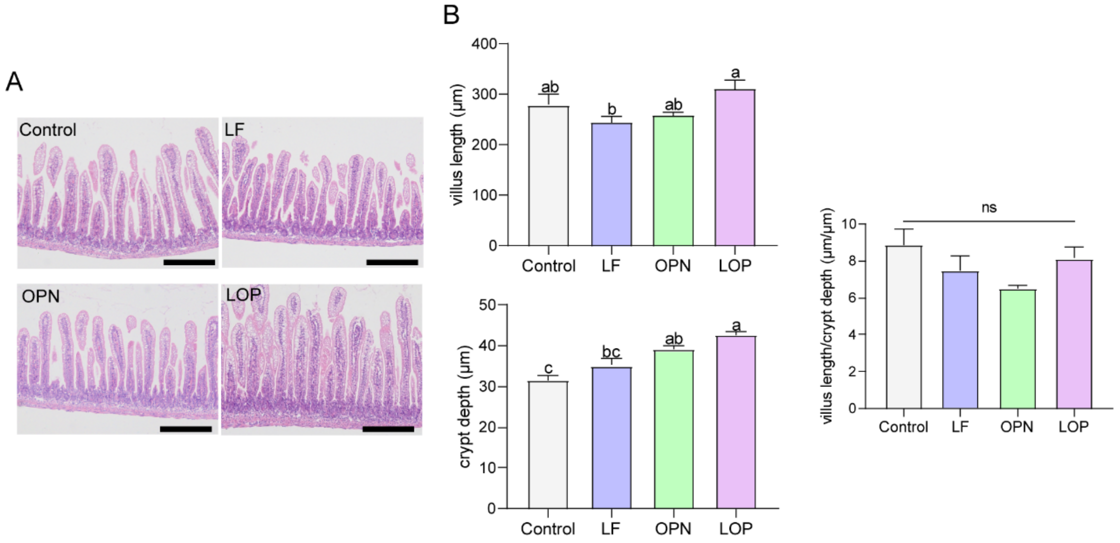

Figure S1. The combined effects of LF and OPN on intestinal structure in 14-day-old mice. (A) Representative images of HE-stained jejunal epithelium (10×, scale bar = 200 μm). (B) Villus length, crypt depth, and the ratio of villus length to crypt depth. Data were shown as mean ± SD, n = 6. Statistical significance of the differences was determined using one-way ANOVA. Different superscript letters (a, b, c) indicated statistically significant differences ($p < 0.05$).

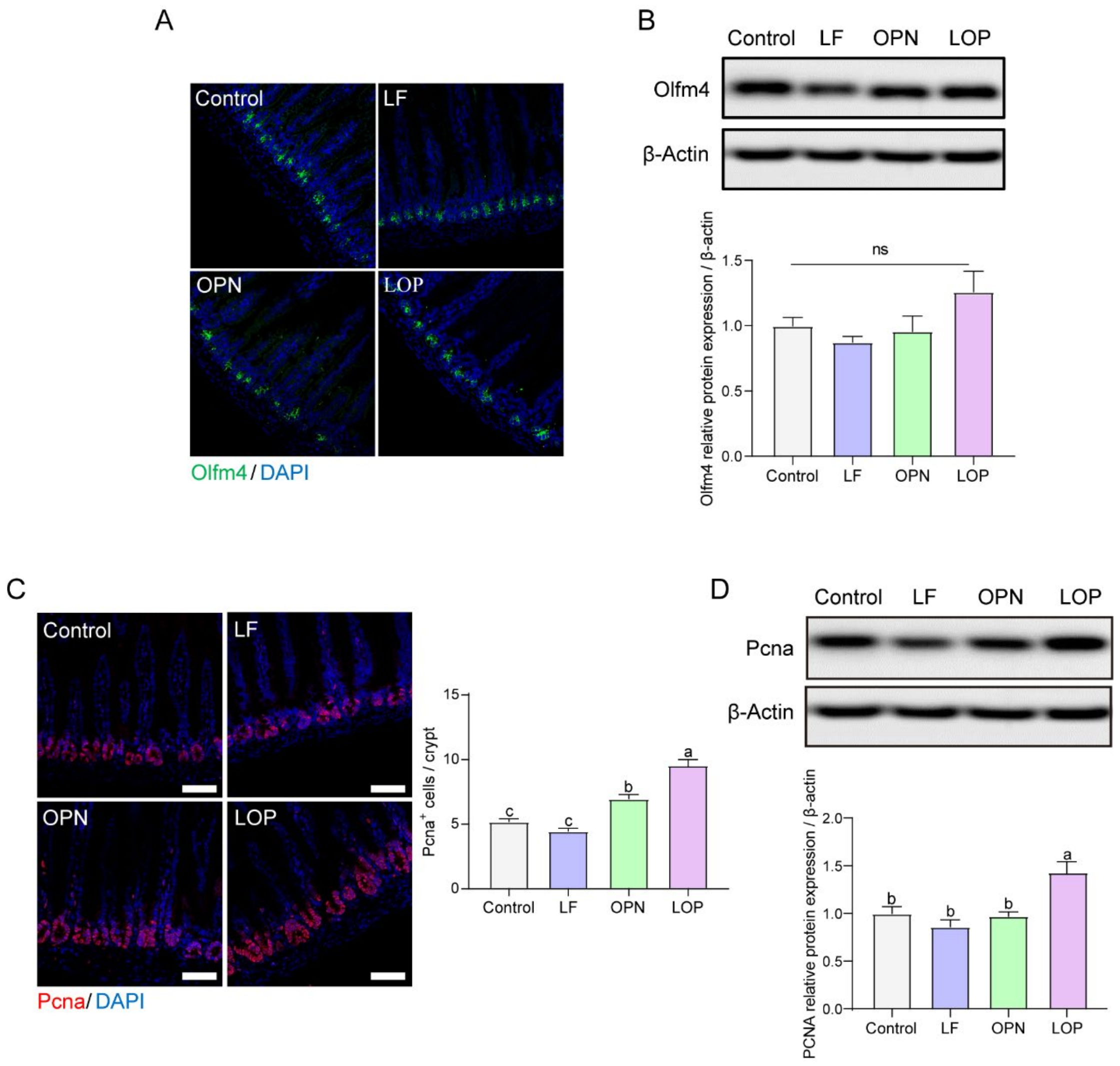

Figure S2. Effects of LF in combination with OPN on intestinal stem cell activation in 14-day-old mice. (A) Representative fluorescence images of intestinal stem cells ($Olfm4^+$) (10×, scale bar = 100 μm). (B) Western blot analysis of Olfm4 expression in crypts. (C) Representative fluorescence images and quantitative analysis of transit-amplifying cells ($Pcna^+$) in crypts (20×, scale bar = 100 μm). (D) Western blot analysis of Pcna expression in crypts. Data were shown as mean ± SD, n = 3. The significance of differences among multiple groups was evaluated using one-way ANOVA. a, b, and c represent significant differences ($p < 0.05$).

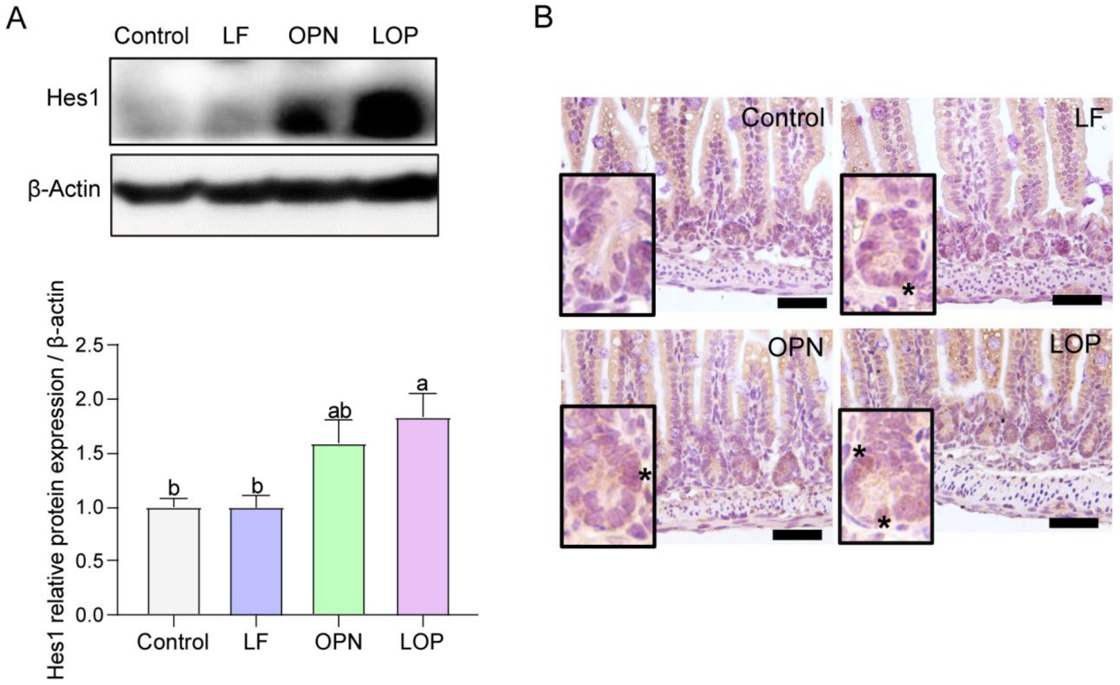

Figure S3. Effects of LF in combination with OPN on HES1 expression in 14-day-old mice. (A) Western blot analysis of Hes1 protein expression. (B) Representative images of the target protein Hes1 in the jejunum. The asterisk "*" indicates the cells with "hes1" (20×, scale bar = 100 μm). Data were shown as mean ± SD, n = 3. The significance of differences among multiple groups was evaluated using one-way ANOVA. a, b, and c represent significant differences ($p < 0.05$).

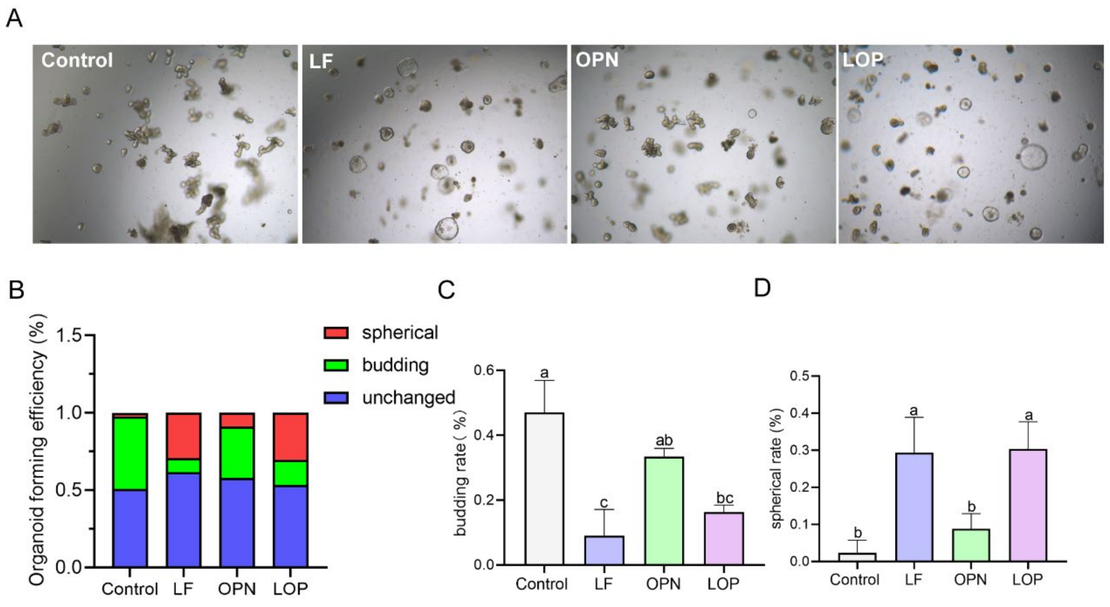

Figure S4. The effects of LF, OPN, and LOP on organoid morphology after in vitro digestion. (A) Representative images of intestinal organoids treated with digested proteins for 3 days. (B) Organoid forming efficiency. (C) Budding rate. (D) spherical rate. Data were shown as mean ± SD, n = 3. The significance of differences among multiple groups was evaluated using one-way ANOVA. a, b, and c represent significant differences ($p < 0.05$).
